# The prevalence and risk factors of sarcopenia in patients with type 2 diabetes mellitus: a systematic review and meta-analysis

**DOI:** 10.1186/s13098-021-00707-7

**Published:** 2021-09-03

**Authors:** Yaqin Ai, Ruoxin Xu, Lingping Liu

**Affiliations:** 1 Medical Department, The Fourth Affiliated Hospital of Nanchang University, Nanchang, 330000 Jiangxi China; 2 Jiangxi Medical College, Nanchang University, No. 461 Bayi Road, Donghu District, Nanchang, 330006 Jiangxi China; 3 Department of Endocrinology, Zhuhai people’s hospital (Zhuhai hospital affiliated of Jinan University), Zhuhai, 519000 Guangdong China

**Keywords:** Diabetes, Sarcopenia, Prevalence, Risk factors, Meta-analysis

## Abstract

**Background:**

Sarcopenia was a frequent chronic complication in patients with type 2 diabetes mellitus (T2DM), and previous evidence showed conflicting results regarding the prevalence and risk factors of sarcopenia in T2DM. In the current study, we aimed at systematically exploring the prevalence and risk factors of sarcopenia in patients with T2DM.

**Methods:**

PubMed, Embase, and Cochrane Central Register of Controlled Trials were systematically searched to identify observational studies which investigated the prevalence and risk factors of sarcopenia in patients with T2DM. The quality of individual included studies was evaluated using The Newcastle–Ottawa scale. Pooled effects regarding prevalence and associated factors were calculated using random-effects models. The potential publication bias was assessed via funnel plot and Egger test.

**Results:**

Twenty-eight studies involving 16,800 patients were included in our meta-analysis. The pooled prevalence of sarcopenia in patients with T2DM was 18% (95% CI 0.15–0.22; I^2^ = 97.4%). The pooled results showed that elder age (OR 4.73; 95% CI 4.30–5.19; I^2^ = 85.6%), male gender, chronic hyperglycemia (higher HbA1c) (OR 1.16; 95% CI 1.05–2.47; I^2^ = 99.2%) and osteoporosis (OR 1.16; 95% CI 1.05–2.47; I^2^ = 99.2%) was predictors for sarcopenia, whereas patients with lower BMI (OR 1.16; 95% CI 1.05–2.47; I^2^ = 99.2%) and metformin administrations (OR 1.16; 95% CI 1.05–2.47; I^2^ = 99.2%) were not prone to get sarcopenia. The funnel plot and statistical tests showed no obvious publication bias.

**Conclusions:**

Sarcopenia was frequent in T2DM patients. Elder age, male gender and chronic hyperglycemia, Osteoporosis were significant risk factors for Sarcopenia. Lower BMI and metformin administrations were associated with lower risk of sarcopenia.

**Supplementary Information:**

The online version contains supplementary material available at 10.1186/s13098-021-00707-7.

## Background

Sarcopenia, an age-related syndrome characterized by progressive and generalized loss of skeletal muscle mass and function, was reported by Irwin Rosenberg in 1989 [1]. Studies showed sarcopenia is associated with poor physical performance, functional impairment, and significantly increased risks of falls, fractures, hospitalization and even death [2, 3]. As age increases, body muscle content gradually decreases, fat tissue gradually increases, and the prevalence of sarcopenia gradually increases. Elderly sarcopenia is an important cause of many adverse events, which significantly increases the risk of various injuries, long-term bed rest and disability, and the risk of disability and death in the elderly, which has a great impact on the quality of life of the elderly [4].

Diabetes mellitus (DM) is a group of metabolic diseases caused by multiple causes, characterized by high blood glucose, which can be caused by the joint action of genetic and environmental factors, and its pathogenesis is relatively complicated and has not been fully elucidated yet [5, 6]. DM is currently one of the highest prevalence rates of chronic non-communicable diseases in the world [7]. According to the epidemiology of diabetes, approximately 387 million adults worldwide suffer from DM, which is estimated to increase to 592 million by 2035 [8].

Researches showed that incidence of sarcopenia was significantly higher among type 2 diabetes mellitus (T2DM), and losing muscle mass and muscle function occurs in the early stage of type 2 diabetes, which declines more significantly with age compared to euglycemic subjects [9, 10]. Korean Sarcopenic Obesity Study (KSOS) reported the incidence of decreased muscle mass in diabetic patients is twice that of euglycemic subjects [11]. Hence, the diagnosis and prevention of senile sarcopenia in patients with T2DM is gradually becoming an important issue in geriatric research. At present, there are few reviews on sarcopenia in this special group of patients with diabetes. Therefore, it is necessary to conduct a meta-analysis on the prevalence and risk factors of sarcopenia in patients with type 2 diabetes. The purpose of this study is to explore the prevention and intervention measures of diabetic myopathy in the elderly, reduce the prevalence rate of sarcopenia, improve the overall health quality of elderly patients with T2DM.

## Methods

The current study was performed according to the Preferred Reporting Items for Systematic reviews and Meta-Analysis (PRISMA) guidelines [12] (showed in Additional file 1: Table S1) and Guidelines for Meta-Analyzes and Systematic Reviews of Observational Studies (MOOSE) [13]. Two reviewers conducted literatures search, data extraction, assessment of quality, and statistical analysis, with inconsistence resolved by a third reviewer. the review was not registered on PROSPERO.

### Literature search

PubMed, Embase, and the Cochrane Library were systematically searched from the inception to December 2020. The eligible studies were identified according to the “PICOS” principle. The search was conducted using these terms, including “sarcopenia”, “type 2 diabetes mellitus”, “T2DM”, “prevalence”, “risk factors” and their variants. Also, we searched the references of the included studies and important reviews for any potential inclusion.

### Inclusion criteria

In the current study, we included observational studies (including cohort studies, case–control studies, or cross-sectional studies) which investigated the prevalence and risk factors of sarcopenia in patients with T2DM. Only studies published in full-text form were considered for inclusion. We merely included original studies published in English, and other non-English publications were excluded. Meanwhile, other studies included letters, comments, and review articles were excluded from the current meta-analysis.

### Data extraction

A pre-designed Excel table was used to extract the following data: first author, publication year, study period, country, case number, the number of patients with sarcopenia, sarcopenia definition, risk factors of sarcopenia, and study design. In the current meta-analysis, the primary outcome is the prevalence of sarcopenia in patients with T2DM. The secondary outcome is the relevant risk factors of sarcopenia in patients with T2DM. Only odds ratios (ORs) with confidence intervals (CIs) on the multivariate analysis in individual included studies were extracted for meta-analysis, while univariate risk factors were excluded.

### Assessment of quality

The quality of individual included studies was evaluated using Newcastle–Ottawa Scale (NOS) under the recommendation of the Cochrane Collaboration [14]. The NOS score involves three domains: selection of participants, comparability of study groups, and ascertainment of outcome or exposure. The total NOS score was designated as nine scores and studies with scores ≥ 7 was defined to be high-quality.

### Statistical analysis

Binary variables were measured by odds ratios (ORs) with 95% CIs and continuous variables were calculated by weighted mean differences (WDs) with 95% CIs. Statistical heterogeneity was quantified using I^2^ statistic and we considered significant heterogeneity if the I^2^ > 50%. Heterogeneity was presented by Cochran’s Q test and p value was less than 0.05, or if the I^2^ statistic was greater than 50% [15]. Random-effects models were used to pool outcomes for the high heterogeneity. We considered a two-side P < 0.05 to be statistical significance. Meta-analysis was undertaken where two or more studies examined the same risk factor in a comparable manner (numerical data available and comparable units of measurement). All the above statistical analysis was conducted using STATA 12.0 (Stata Corporation, College Station, TX, USA).

### Subgroup analysis

In order to explore potential heterogeneity across studies, subgroup analysis was conducted by age, article type, sample size, NOS score, diagnostic criterion, definition of sarcopenia, diagnostic modality and region.

### Publication bias and sensitivity analysis

Sensitivity analysis was performed to test robustness of pooled result of this review by omitting one study in each turn. The publication bias was assessed by inspecting funnel plots qualitatively and Begg–Mazumdar rank continuity correlation and Egger’s regression quantitatively [16, 17].

## Results

### Study characteristics

**Fig. 1:**
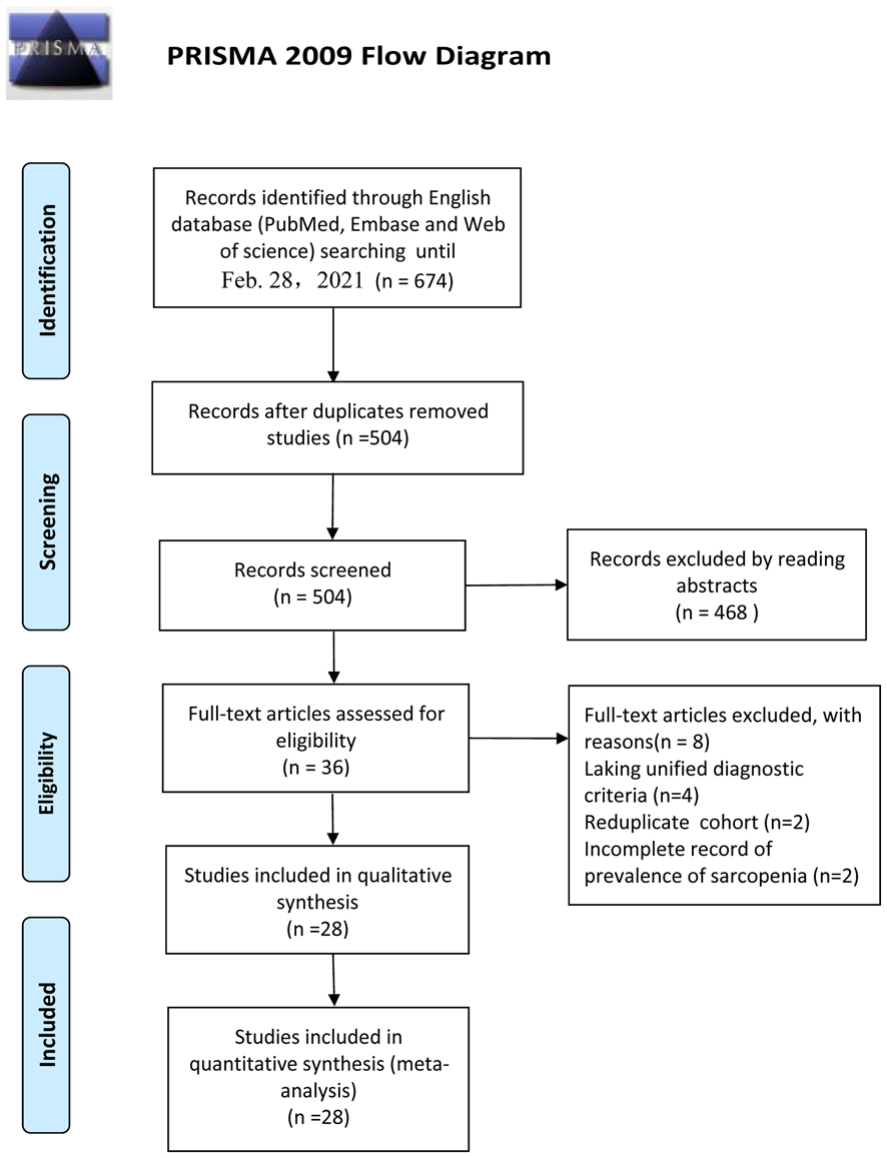
PRISMA flow diagram of literature selection

As shown in Fig. 1, 674 articles were systematically researched from PubMed, Embase, and Cochrane Central Register of Controlled Trials and after removing duplicates and irrelevant records, remaining 504 articles were further reviewed through title and abstract and 36 articles were assessed for eligibility by scanning full text. Of these 36 articles, two studies [18, 19] included duplicated cohort, four studies [11, 20–22] were excluded for lacking unified diagnostic criteria and two studies [23, 24] were excluded for an incomplete record of prevalence of sarcopenia. Finally, 28 articles were deemed suitable to include in the meta-analysis after full-text screening [25–53] (Shown in Table 1).

**Table 1:** General characteristics of the included studies

Author (et al.) , year	Country	Study design	Patients with T2DM (n)	Age (years)^a^	Sex ratio (M/F)	Duration of T2DM (years)^a^	Patients with sarcopenia (n)	Prevalence ofsarcopenia (%)	Diagnostic criterion	Definition of sarcopenia	Diagnostic modality
Ken 2021	Japan	The MUSCLES-DM longitudinal study	588	70.0 ± 8.9	346/242	NA	37	6.3	LSMI	AWGS	BIA
Kang 2021	Korea	The Korean frailty and aging cohort study (KFACS)	2403	76.0 ± 3.9	1134/1269	NA	353	14.7	LMM + LMS	AWGS/FISH	DEXA
Takahashi 2020	Japan	KAMOGAWA-DM Cohort Study (prospective cohort study)	351	66.6 ± 10.6	192/159	14.1 ± 10.0	58 (W: 22, M:46)	16.5	LMM + LMS	AWGS	BIA
Sung 2020	Korea	Observational longitudinal study	309	62.7 ± 10.5	215/94	NA	75	24.3	LSMI	EWGSOP	CT
Seo 2020	Korea	Cross-sectional study	4210	57.4 ± 10.8	2160/2050	7.8 ± 7.3	1240	29.5	LSMI	KNHANES	BIA
Pechmann 2020	Brazil	Cross-sectional study	177	65.6 ± 8.6	63/114	15.4 ± 8.2	23	12.9	LMM	FISH	DEXA
Nakanishi 2020	Japan	Cross-sectional study	1137	73.7 ± 6.3	661/476	17.2 ± 10.3	142 (W: 85, M: 57)	12.5	LMM + LMS	AWGS	DEXA
Mori 2020	Japan	Multi-institutional joint cross-sectional study	645	72.4 ± 7.9	390/255	16.6 ± 11.5	76 (W: 29, M: 47)	11.8	LSMI	AWGS	BIA
Beretta 2020	Brazil	Prospective cohort study	306	71.35 ± 6.45	NA	NA	144	47.1	LMM + LMS	EWGSOP	inextensible tape measure
Sazlina 2020	Malaysia	The MUSCLES-DM longitudinal study	506	67.6 ± 6.8	202/304	NA	144 (W: 75, M: 69)	28.5	LMM	AWGS	BIA
Jung 2020	Korea	Cross-sectional study	102	55. 9 ± 9.8	65/37	9 (2.4–5.3)	12 (W: 4, M: 8)	11.8	LMM	AWGS	BIA
Gorial 2020	Iraq	Case–control study	65	57.0 ± 7.7	23/42	7.2 ± 6.0	10	15.4	LMM + LMS	EWGSOP	DEXA
Mauren 2020	Brazil	Cross-sectional study	484	68.3 ± 5.6	224/260	14(8–22)	58 (EWGSOP1:41EWGSOP2:17)	12.0	LMM + LMS	EWGSOP1/2	BIA
Cui 2020	China	Cross-sectional study	132	73.5 (68–77.25)	59/73	14.0 (5.0–22.0)	38	28.8	LSMI + LMM	AWGS	DEXA
Chen 2020	China	Cross-sectional observational study	1732	NA	654/1078	NA	148 (W: 117, M: 31)	10.37	LMM + LMS	AWGS	BIA
Yanagita 2019	Japan	Retrospective cohort study	108	76.2 ± 67.3	47/61	14.3 ± 12.1	38 (W: 25, M: 13)	35.2	LMM + LMS	AWGS	BIA
Okamura 2019	Japan	KAMOGAWA-DM cohort study(prospective cohort study)	433	65.4 ± 11.1	236/197	10.3 ± 10.1	32 (W: 16, M: 16)	7.4	LSMI	AWGS	DEXA
Ken 2019	Japan	the MUSCLES-DM longitudinal study	746	69.9 ± 9.0	450/296	15.8 ± 11.6	52 (W: 20, M: 32)	7.0	LSMI	AWGS	BIA
Kaji 2019	Japan	The KAMOGAWA-DM cohort study	144	71.4 ± 6.7	82/62	15.2 ± 9.4	17 (W: 5, M: 12)	11.8	LSMI	AWGS	BIA
Noriko 2019	Japan	Cross-sectional study	138	75.0 ± 5.3	72/66	15.3 ± 10.8	17	12.3	LMM + LMS	AWGS	DEXA
Fung 2019	Singapore	Cross-sectional study	387	68.3 ± 5.7	206/181	NA	106 (W: 61, M: 45)	27.4	LMM + LMS	AWGS	BIA
Trierweiler 2018	Brazil	Cross-sectional study	83	65.84 ± 8.82	24/59	15.55 ± 8.67	13	15.7	LSMI	FISH	DEXA
Murai 2018	Japan	Cross-sectional study	183	64.7 ± 12.6	126/57	9 (3–21)	41 (W: 15, M: 26)	22.0	LMM	AWGS	BIA
Hashimoto 2018	Japan	A cross-sectional study of the KAMOGAWA-DM cohort study	146	72.6 ± 5.9	86/60	15.3 ± 9.3	21 (W: 5, M: 16)	14.4	LSMI	AWGS	BIA
Murata 2017	Japan	Cross-sectional study	288	73.3 ± 6.1	151/137	17.0 ± 10.0	44 (W: 21, M: 23)	15.3	LSMI	AWGS	BIA
Bouchi 2017	Japan	Cross-sectional study	238	64 ± 12	145/93	8 (3–14)	42	17.6	LMM + LMS	AWGS	DEXA
Wang 2016	China	Cross-sectional study	236	68.4 ± 7.9	116/120	9.31 ± 7.32	35 (W: 15, M: 20)	14.8	LSMI	AWGS	BIA
Tanaka 2015	Japan	Cross-sectional study	191	60.2 ± 12.5	191/0	9.8 ± 8.7	85 (W: 0, M: 85)	44.5	LSMI	EWGSOP	DEXA

*T2DM* type 2 diabetes mellitus, *NA* not available, DEXA dual-energy X ray absorptiometry, *BIA* bioelectrical impedance analysis, *CT* computed tomography, *LMM* low muscle mass, *LMS* low muscle strength, *LSMI* low skeletal muscle mass index, *AWGS* The Asian Working Group for Sarcopenia, *FISH* The Foundation for the National Institutes of Health, *EWGSOP* The European Working Group on Sarcopenia in Older People, *KNHANES* The Korea National Health and Nutrition Examination Study

^a^Data were shown as mean ± standard deviation (SD) or Median (IQR)

Totally 28 studies contained 16,634 patients with T2DM were included into this meta-analysis and baseline characteristics of the included studies were summarized in Table 1. These included studies with mean age ranging from 55.9 to 76.2 and sample size from 65 to 4210 were published between 2015 and 2021, within which, 14 studies were performed in Japan, four in Korea, four in Brazil, three in China and one each in Iraq, Malaysia and Singapore. Diagnostic criterion of sarcopenia in four studies were defined according to low muscle mass (LMM), 11 were low muscle mass (LMM) + low muscle strength (LMS) and 13 were low skeletal muscle mass index (LSMI). About the definition of sarcopenia, the Asian Working Group for Sarcopenia (AWGS) was applied in 19 studies, the European Working Group on Sarcopenia in Older People (EWGSOP) was used in four studies, the Foundation for the National Institutes of Health (FISH) was used in two studies and other diagnostic criterion were applied in three studies. And regarding the diagnostic modality, bioelectrical impedance analysis (BIA) was applied as measuring instrument in 16 studies, Dual-energy X-ray absorptiometry (DEXA) was applied in 10 studies, computed tomography (CT) was used in one study and inextensible tape measure was used in one study.

### Quality of evidence

The quality and level of evidence of included studies was summarized in Table 2, according to Wells et al. [14]. The scores of included studies ranged from 5 to 9. No studies were excluded based on methodological quality. The scores were range from 5 to 7, which showed the quality of included studies was low, while the NOS score was over 7 showed the quality of included studies was high.

**Table 2:** Methodological quality of included studies based on the Newcastle–Ottawa scale

Studies (n = 28)	Selection(0–4stars)	Comparability (0–2 stars)	Outcome (0–3 stars)^a,b^	Total NOS score (0–9)
Tanaka 2015	***	*	**	6
Wang 2016	***	*	*	5
Murata 2017	***	*	**	6
Bouchi 2017	**	*	**	5
Trierweiler 2018	***	**	**	7
Murai 2018	****	*	***	8
Hashimoto 2018	***	*	**	6
Yanagita 2019	****	**	**	8
Okamura 2019	****	*	**	7
Ken 2019	****	**	**	8
Kaji 2019	****	*	**	7
Noriko 2019	****	*	**	7
Fung 2019	****	*	***	8
Takahashi 2020	****	**	***	9
Sung 2020	**	**	**	6
Seo 2020	**	*	***	6
Pechmann 2020	****	**	**	8
Nakanishi 2020	***	**	**	8
Mori 2020	****	**	**	8
Beretta 2020	***	*	*	5
Sazlina 2020	***	*	*	5
Jung 2020	****	**	***	9
Gorial 2020	**	*	**	5
Mauren 2020	****	**	**	8
Cui 2020	****	*	**	7
Chen 2020	***	*	**	6
Ken 2021	****	**	***	9
Kang 2021	****	**	**	8

A study can be awarded a maximum of one star for each numbered item within the Selection and Exposure categories and maximum of two stars can be given for comparability

^a^A cohort study with a follow-up time > 6 months was awarded one star

^b^A cohort study with a follow-up rate > 75% was awarded one star

### Prevalence of sarcopenia and subgroup analysis

**Fig. 2:**
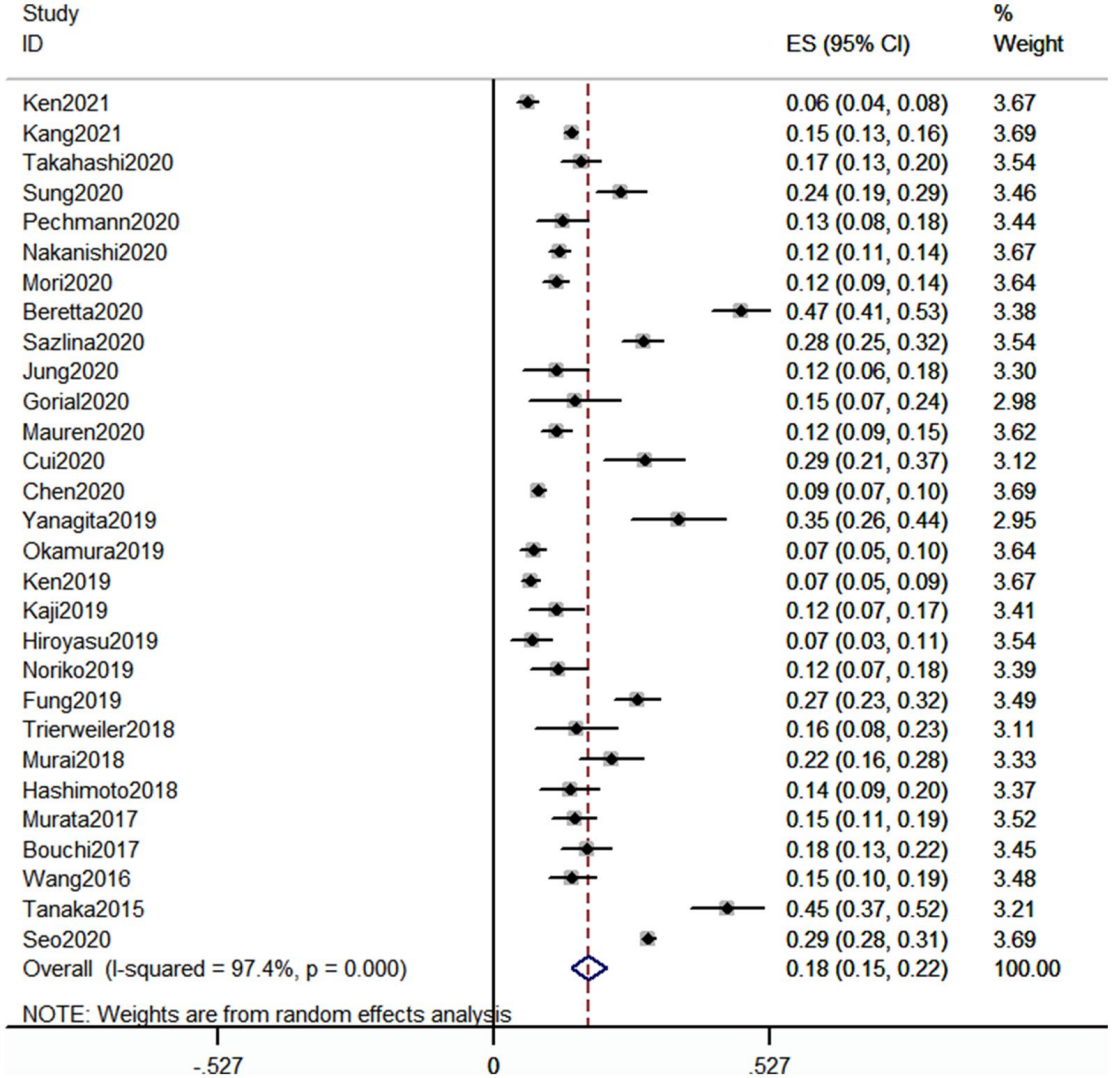
Meta-analysis of prevalence of sarcopenia in patients with type 2 diabetes mellitus

Twenty eight studies reported the prevalence of sarcopenia in patients with T2DM (presenting in Fig. 2). The prevalence of sarcopenia was reported range from 6.3 to 47.1%. And the pooled prevalence of sarcopenia was 18% (95 CI 15–22%), with severe heterogeneity (I^2^ = 97.4%, P < 0.01).

Subgroup analyzes were performed to explore the potential source of heterogeneity across studies according to age, article type, sample size, NOS score, diagnostic criterion, definition of sarcopenia, diagnostic modality and region, as show in Additional file 2: Table S2. 16 studies with mean age ≥ 70 reported the rate of sarcopenia was 19% (95% CI 14–25%), with evidence of high interstudy heterogeneity (I^2^ = 97.5%; Heterogeneity < 0.001). And 11 studies with mean age < 70 reported the rate of sarcopenia was 18% (95% CI 14–23%), with evidence of high interstudy heterogeneity (I^2^ = 95.7%; Heterogeneity < 0.001). While one study did not report the specific age of included cohort. According to article type, 17 cross-sectional study reported the rate of sarcopenia was 18% (95% CI 13–23%), with evidence of high interstudy heterogeneity (I^2^ = 97.4%; Heterogeneity < 0.001), 6 cohort study reported the rate of sarcopenia was 22% (95% CI 13–30%), with evidence of high interstudy heterogeneity (I^2^ = 97.3%; Heterogeneity < 0.001), four longitudinal studies reported the rate of sarcopenia was 16% (95% CI 7–26%), with evidence of high interstudy heterogeneity (I^2^ = 97.9%; Heterogeneity < 0.001) and one case–control study reported the rate of sarcopenia was 15% (95% CI 7–24%). 14 studies with large sample size ≥ 300 reported the rate of sarcopenia was 19% (95% CI 15–24%), with evidence of high interstudy heterogeneity (I^2^ = 87%; Heterogeneity < 0.001), while 14 studies with small sample size < 300 reported the rate of sarcopenia was 18% (95% CI 13–23%), with evidence of high interstudy heterogeneity (I^2^ = 98.6%; Heterogeneity < 0.001). As for NOS score, 17 studies with a total score of ≥ 8 clearly reported incidence of sarcopenia was 24.0% (95% CI 16–31%), with evidence of high interstudy heterogeneity (I^2^ = 98.4%; Heterogeneity < 0.001), while 11 studies with NOS score of < 8 reported the rate of sarcopenia was 15% (95% CI 12.0–17.0%), with evidence of high interstudy heterogeneity (I^2^ = 91.9%; Heterogeneity < 0.001). 20 studies with the definition of sarcopenia following AWGS reported incidence of sarcopenia was 16.0% (95% CI 13–18%), with evidence of high interstudy heterogeneity (I^2^ = 93.6%; Heterogeneity < 0.001), five studies following EWGSOP reported the rate of sarcopenia was 29.0% (95% CI 14–44%), with evidence of high interstudy heterogeneity (I^2^ = 97.6%; Heterogeneity < 0.001), while two studies following FISH reported the rate of sarcopenia was 14.0% (95% CI 10–18%) and one studies which defined sarcopenia according to The Korea National Health and Nutrition Examination Study (KNHANES) reported the rate of sarcopenia was 29% (95% CI 28–31%). What’s more, 13 studies used LSMI as a diagnostic criterion reported the rate of sarcopenia was 18.0% (95% CI 11–24%), with evidence of high interstudy heterogeneity (I^2^ = 98.3%; Heterogeneity < 0.001), 11 studies used both LMM and LMS as a diagnostic criterion reported the rate of sarcopenia was 19.0% (95% CI 15–24%), with evidence of high interstudy heterogeneity (I^2^ = 96.2%; Heterogeneity < 0.001) and four studies used LMM as a diagnostic criterion reported the rate of sarcopenia was 19.0% (95% CI 11–28%), with evidence of high interstudy heterogeneity (I^2^ = 90.7%; Heterogeneity < 0.001). Based on diagnostic modality, eight studies used BIA as a measuring tool reported the rate of sarcopenia was 17.0%(95% CI 12–22%), with evidence of high interstudy heterogeneity(I^2^ = 98.1%; Heterogeneity < 0.001), five studies used DEXA as a measuring tool reported the rate of sarcopenia was 17.0%(95% CI 13–22%), with evidence of high interstudy heterogeneity(I^2^ = 92.5%; Heterogeneity < 0.001), while one study used CT as a measuring tool reported the rate of sarcopenia was 47.0%(95% CI 41–53%) and one study used inextensible tape measure as a measuring tool reported the rate of sarcopenia was 24.0%(95% CI 19–29%). 14 studies in the region of Japan reported incidence of sarcopenia was 16%(95% CI 12–19%), with evidence of high interstudy heterogeneity (I^2^ = 93.5%; Heterogeneity < 0.001), and four studies in the region of Brazil reported the rate of sarcopenia was 22.0%(95% CI 6–38%), with evidence of high interstudy heterogeneity (I^2^ = 97.6%; Heterogeneity < 0.001), while three studies in China, four studies in Korea and three studies in other countries report the incidence of sarcopenia was 17%(95% CI 7–26%), 20%(95% CI 10–30%) and 25% (95% CI 19–31%) respectively.

### Sensitivity analysis and publication bias

**Fig. 3:**
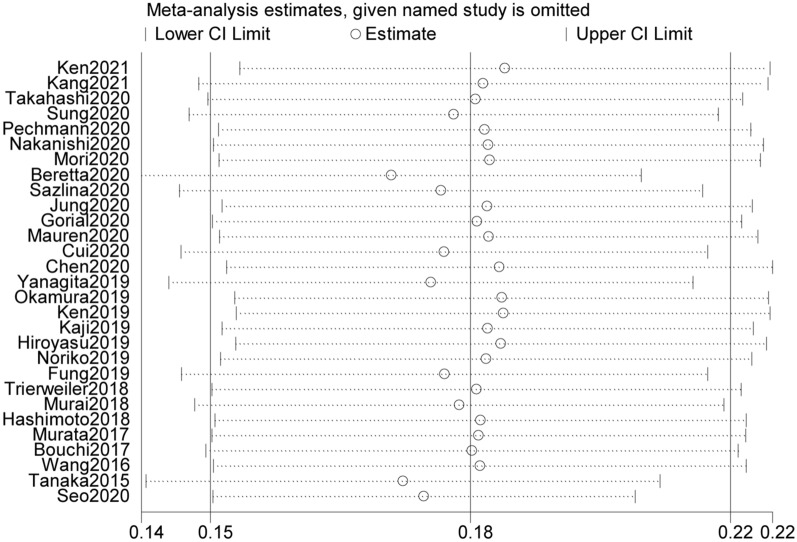
Sensitivity analysis for meta-analysis of prevalence of sarcopenia in patients with type 2 diabetes mellitus

**Fig. 4:**
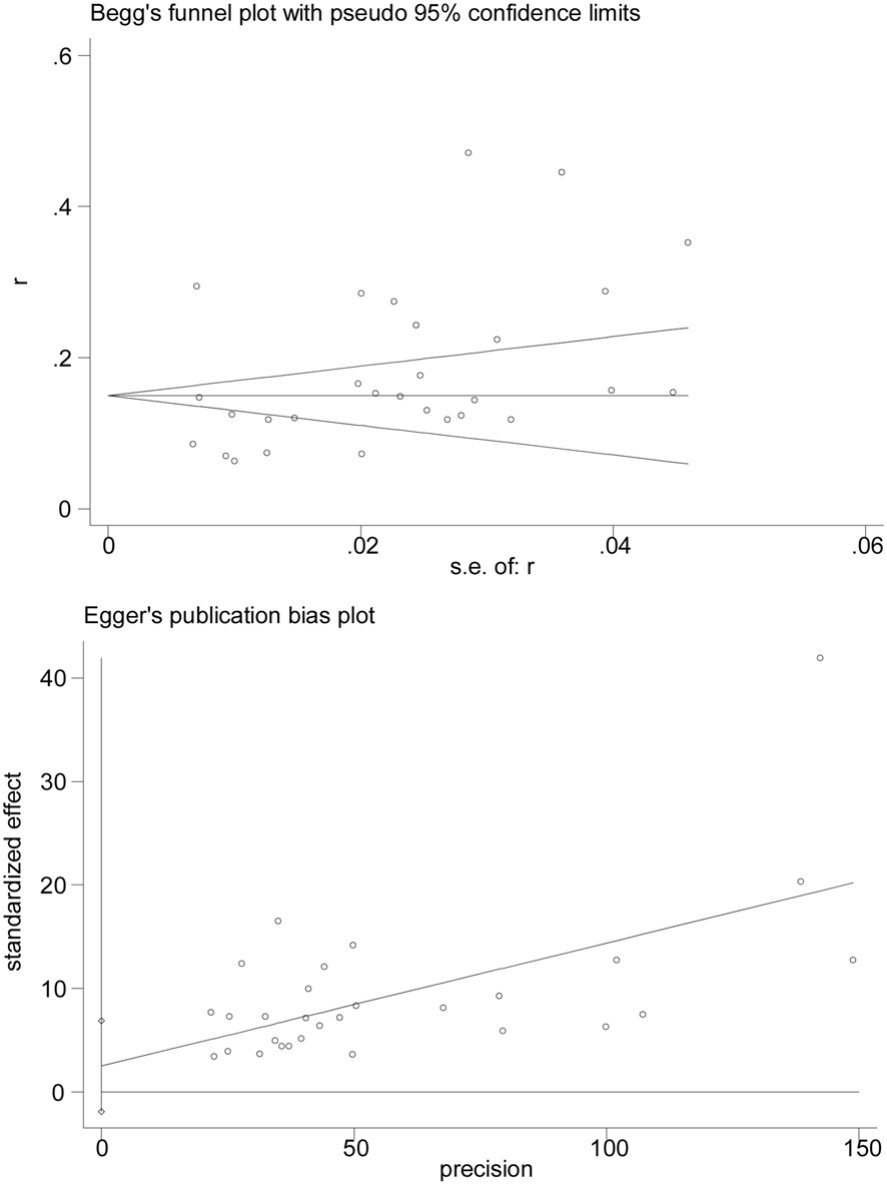
Funnel plots for meta-analysis of prevalence of sarcopenia in patients with diabetes mellitus

We conducted a sensitivity analysis to confirm the robustness of the pooled results. And subgroup analyses and sensitivity analyses (Fig. 3) showed the current pooled evidence was enough credible and robust though there was high heterogeneity among included studies. Meanwhile, publication bias was recognized from visual inspection of funnel plot (Fig. 4) and Begg and Egger tests were carried out (Begg: p = 0.009, Egger: P = 0.284). The funnel plots indicated that P value of Begg test was less than 0.05, which suggested potential publication bias. However, the p value of Egger’s regression intercept was 0.284, this indicated that there was no obvious publication bias. Hence, trimming estimator and Filled analyses were further conducted and the result showed that the pooled estimate data was basically unchanged consistent.

### Risk factors of POD

Risk factors for the prevalence of sarcopenia in patients with T2DM were assessed in this meta-analysis. Risk was assessed by pooling adds ratio (OR) and 95% CI from multivariate analysis and logistic regression with random effects model. Pooled results showed that the prevalence of sarcopenia was statistically significantly associated with five factors: older age (OR 1.16, 95% CI 1.06–1.27), glycosylated hemoglobin A1c (HbA1c) (OR 1.69, 95% CI 1.01–2.83) and osteoporosis (OR 4.79, 95% CI 1.58–14.52) were significant risk factors for sarcopenia in patients with T2DM, while BMI (OR 0.65, 95% CI 0.51–0.82) and metformin (OR 0.37, 95% CI 0.21–0.63) were protective factors for sarcopenia. Meanwhile, sex (male) (OR 1.25, 95% CI 0.79–1.97), diabetic neuropathy (OR 1.53, 95% CI 0.61–3.86), eGFR (OR 0.97, 95% CI 0.93–1.00), duration of diabetes (OR 1.31, 95% CI 0.75–2.27), concurrent hypertension (OR 0.90, 95% CI 0.13–6.06), exercise (OR 0.29, 95% CI 0.07–1.19) and dietary protein intake (OR 0.24, 95% CI 0.03–2.23) were statistically insignificant factors (showed in Additional file 3: Table S3).

## Discussion

The results of present meta-analysis have showed the prevalence of sarcopenia in terms of age, genders or different regions of patients with T2DM was 18% (95% CI 0.15–0.22). Meanwhile, the pooled result showed that different diagnostic criterion, definition of sarcopenia and diagnostic modality influenced the diagnosis rate of sarcopenia. Furthermore, we identified several risk factors, including older age, older age and osteoporosis, while several protective factors, including lower BMI and metformin administrations. The other risk factors for sarcopenia in patients with T2DM, like Sex (male), diabetic neuropathy, eGFR, duration of diabetes, concurrent hypertension Exercise and dietary protein intake, were also explored and were proved to have no association with sarcopenia.

Sarcopenia is an age-related disease with progressive loss of muscle mass and loss of function. Sarcopenia is manifested as decreased muscle content, decreased physical activity, decreased quality of life, and increased risk of falls and death. Diabetes mellitus is a chronic metabolic disease, and the incidence of muscle attenuation in elderly diabetic patients is significantly increased. Sarcopenia has gradually become one of the additional complications of elderly diabetes. Disorders of glucose metabolism increases the risk of decreased muscle mass. Several studies have confirmed that the muscle mass and muscle strength of type 2 diabetic patients decrease more significantly with age than non-diabetic patients [54].

To date, there are four consensus reports on sarcopenia as the theme. They are the 2010 European Working Group Consensus on Elderly Sarcopenia (EWGSOP) [3], the 2010 European Association for Clinical Nutrition and Metabolism Special Interest Group Consensus Report (ESPEN-SIG) [55], the 2011 International Sarcoidosis Conference Working Group Consensus (IWGS) [56] and the 2014 Asian Sarcopenia Working Group Consensus (AWGS) [57]. The current diagnostic methods for sarcopenia are not uniform, and the cut-off points (cut-off points) given in these reports are also slightly different. In our meta-analysis, subgroup analysis based on the definition of sarcopenia showed that the prevalence of sarcopenia varied from 14 to 29%. What’s more, different diagnostic modality, like BIA, DEXA, CT and even inextensible tape measure, were used to assess the muscle mass and strength. With the deepening of understanding, the definition of sarcopenia gradually developed from the early reduction of muscle content as the standard to take into account and even emphasize the decline of muscle function. The Asian Sarcopenia Consensus (AWGS) recommends that the elderly should first be screened for grip strength and gait speed. When there is a drop in grip strength or gait speed, then screen muscle content. If there is a decrease in muscle content, it can be diagnosed as sarcopenia; height correction is recommended. The extremity skeletal muscle index (appendicular skeletal muscle mass index, ASMI) is calculated as the square of the extremity skeletal muscle content (kg)/height (m). The diagnostic cut-off value is lower than the mean of healthy young people of the same sex or over 2 standard deviations or the lowest quintile. If the data of healthy young people cannot be obtained, the recommended diagnostic cut-off value is less than 7.0 kg/m^2^ (DXA method or BIA method) in male, which is less than 5.4 kg/m^2^ (DXA method), 5.7 kg/m^2^ (BIA method) in female. It is recommended to use grip strength to assess muscle strength. The diagnostic cut-off value is the lowest quintile of the same-sex research population, and the recommended cut-off value is less than 26. 0 kg for male, which is less than 18 kg for female. Daily walking speed is used for muscle function, and the diagnostic cut-off value is less than 0.8 m/s.

So far, the mechanism of sarcopenia in patients with type 2 diabetes mellitus is still unclear. There are several possible mechanisms of sarcopenia in patients with type 2 diabetes mellitus: (1) Increased levels of reactive oxygen (ROS) can damage the structure and function of skeletal muscle cells [58–60]; (2) The loss of alpha motor neurons may be the reason for the decrease in muscle mass associated with aging [61]. Type 2 diabetic mellitus patients with neuropathy manifested as: central nervous system complications, peripheral neuropathy, autonomic neuropathy, etc. Electroneurography, electromyography can early detect sensory nerve and motor nerve conduction velocity or conduction disorders [62]. Symptoms such as numbness, pain, dyskinesia, etc. reduce activity, and muscle strength is reduced due to the denutrition of nerves, which leads to the occurrence of sarcopenia; (3) The decrease in protein intake and synthesis, decomposition and consumption too fast lead to the decrease of skeletal muscle mass. The basic treatment of type 2 diabetes is diet control, emphasizing carbohydrate-based treatment [63]. For diabetic patients with normal renal function, the recommended protein intake accounts for 10–15% of the energy supply ratio; for patients with dominant proteinuria, the intake should be limited to 0.8 g per kilogram of body weight per day, which is reduced from GFR Begin to implement a low-protein diet, and the recommended intake is 0.6 g per kilogram of body weight per day [64]. Excessive protein load will increase the burden on the kidneys and further aggravate renal function damage. For patients with diabetic nephropathy, it is necessary to limit the amount of protein intake, thereby reducing the source of muscle fiber synthesis raw materials, and aggravating the occurrence of sarcopenia. In patients with cachexia, a large amount of protein consumption is also an important cause of sarcopenia; (4) Changes in hormone levels: Changes in hormone levels such as estrogen, testosterone, insulin, and adrenocorticotropic hormone (ACTH) lead to changes in the skeletal muscle microenvironment, leading to the occurrence of sarcopenia [65, 66]; (5) Osteoporosis is an important type of diabetic metabolic bone disease. Vitamin D regulates calcium and phosphorus metabolism, maintains normal bone mineral salt levels, and plays a role in the homeostasis of bones and muscles [67]. With age, the expression of vitamin D receptors on the skeletal muscle fiber cell membrane decreases, aggravating vitamin D deficiency in the elderly [68]. Its deficiency is related to increased bone resorption and loss of muscle mass and strength in the elderly [69]. The occurrence of sarcopenia is a process in which type 2 muscle fibers are replaced by type 1 muscle fibers and fat cells. Type 2 muscle fibers play an important role in preventing falls. Vitamin D deficiency can lead to type 2 [70]; (6) Growth hormone (GH) and IGF-1 (Insulin-like growth factor-1, IGF-1) are important regulators of muscle mass. The levels of GH and IGF-1 show a downward trend with age, leading to Decrease in muscle mass and increase in fat mass [71]. In patients with type 2 diabetes, the GH/IGF-1 axis shows an increase in GH and a decrease in IGF-1 [72]. And in this meta-analysis, age, HbA1c, osteoporosis, BMI and metformin were confirmed to be associated with sarcopenia, which was consistent to previous researches.

However, several previous studies reported that the prevalence of sarcopenia in female was higher than that in male [11, 73, 74]. For male, testosterone can increase muscle strength in the elderly, low-dose testosterone can still increase muscle mass and reduce fat mass, while high-dose testosterone can increase muscle mass and muscle strength at the same time [75, 76]. For postmenopausal women, the changes in estrogen levels have an impact on bones and muscles. Estrogen can inhibit bone turnover and prevent bone loss [77]. Estrogen affects skeletal muscle through mechanisms such as improving the level of inflammatory factors in the skeletal muscle environment, resisting protein breakdown, and promoting the proliferation and differentiation of muscle satellite cells [78]. Thus, further high-quality studies were warranted to conduct to explore the relationship between sarcopenia and sex and other potential risk factors.

In addition, several limitations of the present meta-analysis should be taken into account. First, our analysis is based on observational studies and some of them were of inferior quality and a modest sample size. So, heavy weight of smaller trials might affect the authenticity of the results. Second, considerable heterogeneity was observed among the included trials. The targeted population varied greatly. Various diagnostic criteria, definition of sarcopenia and diagnostic modality may cause the heterogeneity and have a potential impact on our results. Finally, it that the exclusion of some missing and unpublished data led to bias in effect size. Finally, it was possible that the exclusion of unpublished data and some missing articles might have led to a bias in the effect.

## Conclusion

In conclusion, sarcopenia was frequent in T2DM patients. Elder age, male gender and chronic hyperglycemia, Osteoporosis were significant risk factors for Sarcopenia. Lower BMI and metformin administrations were associated with lower risk of sarcopenia. These results were robust though the high heterogeneity and lack of high-quality trails, thus the interpretations for those findings should be cautious. Further large-sample and high-quality trails should be carried to demonstrate those results.

## Supplementary Information

**Additional file 1: Table S1.** The Preferred Reporting Items for Systematic reviews and Meta-Analysis (PRISMA) guidelines_2020_checklist.

**Additional file 2: Table S2.** Subgroup analysis of prevalence of sarcopenia in patients with diabetes mellitus.

**Additional file 3: Table S3.** Meta-analysis of risk factors for sarcopenia in patients with diabetes mellitus.

## Data Availability

All data generated or analyzed during this study are included in this published article (and its additional files).
