# Supplementary material for: The prevalence and risk factors of sarcopenia in patients with type 2 diabetes mellitus: a systematic review and meta-analysis
Source: Diabetol Metab Syndr. 2021 Sep 3;13:93. doi: 10.1186/s13098-021-00707-7 (PMC8414692; doi:10.1186/s13098-021-00707-7)
Supplement: Supplementary file 2 — Additional file 2: Table S2. Subgroup analysis of prevalence of sarcopenia in patients with diabetes mellitus. [file 13098_2021_707_MOESM2_ESM.docx]

| **Table 2: Subgroup analysis of prevalence of sarcopenia based on various factors.** | | | |
| --- | --- | --- | --- |
| Outcomes | Number of trials | OR/RR (95% CI) | Heterogeneity, I2 (%) |
| Pooled results | 28 | 1.82(1.27-2.61) | 92.5 |
| Subgroup analyses based age |  |  |  |
| Mean age≥70 | 16 | 0.19(0.14-0.25) | 97.5 |
| Mean age˂70 | 11 | 0.18(0.14-0.23) | 95.7 |
| NR | 1 | 0.09 (0.07-0.10) | - |
| Subgroup analyses based on article type |  |  |  |
| Cross-sectional study | 17 | 0.18 (0.13-0.23) | 97.4 |
| Longitudinal study | 4 | 0.16 (0.07-0.26) | 97.9 |
| Cohort study | 6 | 0.22 (0.13-0.30) | 97.3 |
| Case-control study | 1 | 0.15 (0.07-0.24) |  |
| Subgroup analyses based on number of sample |  |  |  |
| N≥300 | 14 | 0.19 (0.15-0.24) | 87 |
| N˂300 | 14 | 0.18 (0.13-0.23) | 98.6 |
| Subgroup analyses based on quality of included studies (NOS) | | | |
| ≥8 | 17 | 0.24 (0.16-0.31) | 98.4 |
| ˂8 | 11 | 0.15 (0.12-0.17) | 91.9 |
| Subgroup analyses based on diagnostic criterion |  |  |  |
| LMM | 4 | 0.19 (0.11-0.28) | 90.7 |
| LMM + LMS | 11 | 0.19 (0.15-0.24) | 96.2 |
| LSMI | 13 | 0.18 (0.11-0.24) | 98.3 |
| Subgroup analyses based on definition of sarcopenia | | | |
| AWGS | 20 | 0.16 (0.13-0.18) | 93.6 |
| EWGSOP | 5 | 0.29 (0.14-0.44) | 97.6 |
| FISH | 2 | 0.14 (0.10-0.18) | 0 |
| KNHANES | 1 | 0.29 (0.28-0.31) | - |
| Subgroup analyses based on diagnostic modality |  |  |  |
| BIA | 8 | 0.17 (0.12-0.22) | 98.1 |
| DEXA | 5 | 0.17 (0.13-0.22) | 92.5 |
| CT | 1 | 0.47 (0.41-0.53) | - |
| inextensible tape measure | 1 | 0.24 (0.19-0.29) | - |
| Subgroup analyses based on rergion |  |  |  |
| Japan | 14 | 0.16 (0.12-0.19) | 93.5 |
| Brazil | 4 | 0.22 (0.06-0.38) | 97.6 |
| China | 3 | 0.17 (0.07-0.26) | 93.6 |
| Korea | 4 | 0.20 (0.10-0.30) | 98.7 |
| Others | 3 | 0.25 (0.19-0.31) | 72.4 |
| OR= odds ratio; RR= relative ratio; CI= confidence interval; Hb= hemoglobin; NOS= Newcastle-Ottawa Scale; NR= not report.DEXA: Dual-energy X ray absorptiometry; BIA: bioelectrical impedance analysis；CT: computed tomography; LMM: low muscle mass; LMS: low muscle strength; LSMI: low skeletal muscle mass index; AWGS: the Asian Working Group for Sarcopenia; FISH: the Foundation for the National Institutes of Health; EWGSOP: the European Working Group on Sarcopenia in Older People; KNHANES: The Korea National Health and Nutrition Examination Study. | | | |
