# Supplementary material for: The prevalence and risk factors of sarcopenia in patients with type 2 diabetes mellitus: a systematic review and meta-analysis
Source: Diabetol Metab Syndr. 2021 Sep 3;13:93. doi: 10.1186/s13098-021-00707-7 (PMC8414692; doi:10.1186/s13098-021-00707-7)
Supplement: Supplementary file 3 — Additional file 3: Table S3. Meta-analysis of risk factors for sarcopenia in patients with diabetes mellitus. [file 13098_2021_707_MOESM3_ESM.docx]

| **Table 3: Meta-analysis of Risk factors for sarcopenia** | | | |
| --- | --- | --- | --- |
|  | Number of trials | Pooled OR (95% CI) | I2 (%) |
| Old age | 10 | 1.16（1.06-1.27） | 85.6 |
| Sex (male) | 8 | 1.25（0.79-1.97） | 83.3 |
| BMI | 12 | 0.65（0.51-0.82） | 95.4 |
| HbA1c | 5 | 1.69（1.01-2.83） | 44.8 |
| Osteoporosis | 2 | 4.79（1.58-14.52） | 16.3 |
| Diabetic neuropathy | 2 | 1.53（0.61-3.86） | 77.2 |
| eGFR | 2 | 0.97（0.93-1.00） | 0.0 |
| Duration of diabetes | 2 | 1.31（0.75-2.27） | 79.0 |
| Concurrent hypertension | 3 | 0.90（0.13-6.06） | 79.7 |
| exercise | 3 | 0.29（0.07-1.19） | 86.5 |
| Metformin | 2 | 0.37（0.21-0.63） | 0.0 |
| Dietary protein intake | 2 | 0.24（0.03-2.23） | 76.0 |
| OR= odds rate; CI= confident interval; BMI= Body Mass Index; HbA1c= glycated hemoglobin; eGFR= estimated glomerular filtration rate. | | | |
